# Data Report: Educational pathway addressing food and nutrition in amyotrophic lateral sclerosis on the AVASUS platform

**DOI:** 10.3389/fdgth.2024.1476293

**Published:** 2025-01-06

**Authors:** Karla M. D. Coutinho, Felipe Fernandes, Kelson C. Medeiros, Karilany D. Coutinho, Aline de Pinho Dias, Ricardo A. de M. Valentim, Lucia Leite-Lais, Kenio Costa Lima

**Affiliations:** ^1^ Health Sciences Graduate Program, Federal University of Rio Grande do Norte, Natal, Brazil; ^2^ Laboratory for Technological Innovation in Health (LAIS), Federal University of Rio Grande do Norte, Natal, Brazil; ^3^ Federal Institute of Education, Science and Technology of Rio Grande do Norte, Natal, Rio Grande do Norte, Brazil; ^4^ Health Management and Innovation Graduate Program, Federal University of Rio Grande do Norte, Natal, Brazil; ^5^ Department of Biomedical Engineering, Federal University of Rio Grande do Norte, Natal, Brazil; ^6^ Department of Nutrition, Federal University of Rio Grande do Norte, Natal, Brazil

**Keywords:** amyotrophic lateral sclerosis, food and nutrition education, nutrition, distance education, internet, online courses

## 1 Introduction

Amyotrophic lateral sclerosis (ALS) is a rare neuromuscular degenerative disease characterized by the progressive loss of motor neurons, which leads to the gradual atrophy of skeletal and respiratory muscles (1–3). The etiology of ALS is multifactorial (2). The disease typically begins in a specific region, affecting the upper or lower limbs, bulbar areas, or respiratory system. As it progresses, it results in muscle paralysis and/or respiratory dysfunction (4).

Malnutrition is common among ALS patients, with prevalence ranging from 16% to 55% at the time of diagnosis, increasing with disease progression (5). Weight loss and malnutrition are associated with faster disease progression, higher risk of complications, decline in quality of life, and reduced survival (5–7). Since nutritional status and metabolic balance impact the prognosis of ALS patients (8, 9), integrating nutritional care into the multidisciplinary treatment of ALS is imperative (10). In other words, multidisciplinary care strategies that focus on managing symptoms and improving nutritional status can enhance the quality of life and extend survival for ALS patients (11, 12).

Caregivers of ALS patients play a vital and continuous role in patient care and should be considered integral members of the multidisciplinary team. Both health professionals and caregivers often require specialized training to effectively support ALS patients. In this context, health education is essential because it not only promotes health and prevents complications but also fosters engaged individuals who are equipped with the skills for self-care and autonomy (13, 14).

In health education, the use of technology-mediated interactions aligns with the growing role of Information and Communication Technologies (ICT) in creating innovative and dynamic educational approaches. These initiatives have enhanced the connection between communication, science, and society (15, 16).

The Virtual Learning Environment of the Brazilian Health System (AVASUS) is a free and open technology-mediated educational platform of Brazil's Ministry of Health. AVASUS was developed by the Laboratory for Technological Innovation in Health (LAIS) at the Federal University of Rio Grande do Norte (UFRN), through technical and scientific cooperation (17–20). The platform offers professional qualification and refresher courses within the Brazilian National Health System (SUS), designed to promote, support, and enhance continuing health education (18, 21). Additionally, it serves as a key tool for fostering resilience and responsiveness within the health system. AVASUS is accessible free of charge to health professionals, students, and the general public. Recent data show that AVASUS has over 1.2 million users, more than 3.3 million enrollments, and 424 active courses (22), making it the third-largest public health education platform globally. Studies have demonstrated that AVASUS fosters massive health professional training in various areas of health (17, 18, 23, 24).

In light of this, this study aimed to structure and share a database capable of serving as a basis for studies interested in this type of information for health education. It provides a demographic characterization of students enrolled in the educational pathway addressing food and nutrition for people living with ALS. Additionally, it includes descriptive information about the courses within this pathway, in which 20,967 enrollments from the five regions of Brazil and abroad have enrolled.

## 2 Materials and methods

### 2.1 Study design and participant

This article presents a Data Report which provides a descriptive analysis of the educational pathway focused on food and nutrition for people living with ALS. The pathway consists of four self-learning courses delivered in a Massive Open Online Course (MOOC) format through AVASUS (25–27). As of June 5, 2024, these courses had amassed 14,450 students and 20,967 enrollments, considering that one student could enroll in up to four courses. The study covers the period from June 1, 2021, to June 5, 2024.

### 2.2 Data acquisition

The descriptive analysis data was elicited from four sources: (i) Virtual Learning Environment of the Brazilian Health System (AVASUS); (ii) National Register of Health Facilities (CNES); (iii) Brazilian Classification of Occupations (CBO); (iv) Brazilian Institute of Geography and Statistics (IBGE). The data was duly anonymized, integrated, and made available through the following public repository: https://doi.org/10.5281/zenodo.12811144. Such data do not allow nor characterize an experimental study with human beings, precluding the need for approval by a research ethics committee, under Resolution No. 510/2016 and No. 674/2022 by CEP/CONEP in Brazil (28, 29).

AVASUS was the primary data source for this study, supplying the majority of the data for descriptive analysis. Information was collected from 20,967 enrollments in the courses. For each student, a set of 340 characteristics or attributes was gathered. The main characteristics analyzed included the student's gender, CNES information, certificates of completion issued, the Brazilian region where the course was taken, CBO details, and the results of both quantitative and qualitative course evaluations completed by each student.

The data collection period extended from June 1, 2021, when the first course was launched, to June 5, 2024. Notably, the courses in the educational pathway remain available on AVASUS, continuously allowing new enrollments from health students, professionals, and the general public. Data on the Brazilian population and regions were obtained from the IBGE's 2022 demographic census. The dataset for this report includes information on all students and their attributes, enabling a comprehensive demographic characterization. Additionally, students’ perspectives and evaluations were collected through a Likert scale and a comment box available on AVASUS at the end of each course.

### 2.3 Data processing

Data engineering tasks were carried out in order to share information with the scientific community. A systematic approach was used to clean, transform, and validate the data in multiple stages for descriptive analysis. The data analysis process included the following steps: (i) data quality assessment; (ii) data integration and standardization; (iii) feature extraction; and (iv) feature selection. All these steps were performed in a Python 3.10.12 environment using auxiliary libraries such as NumPy, Pandas, Matplotlib, Seaborn, and Enelvo.

In step (i), the dataset was inspected to identify instances with missing, inconsistent, or noisy data. During step (ii), it was necessary to retrieve the CBO codes for participants registered through CNES. These codes were linked to their respective records and incorporated into the main dataset as a new attribute. Participants without a formal professional affiliation or CBO code were labeled as “individuals with no formal affiliation”. Additionally, for the attribute related to students’ gender, standardization of nomenclature was performed, resulting in the categories: Female, Male, and Not Reported.

In step (iii), we developed features related to the region and the descriptive classification of students’ professions. Using the CBO code and the official CBO data source for Brazil (30), the team decoded the CBO codes and incorporated the corresponding occupation names into the dataset. To address variations and minimize discrepancies among synonymous occupations, we applied regular expressions. For example, different descriptions derived from the medical field, i.e., specialist doctors, were consolidated into a single category labeled “Doctor.”

Lastly, the region attribute was derived from the student's Federative Unity (FU) information found in the AVASUS dataset. This attribute was used to categorize students into one of Brazil's five major regions (North, Northeast, Central-West, Southeast, and South), based on Brazil's political-administrative regional divisions (31). In step (iv), we identified the key elements necessary for the descriptive analysis of this study. This stage involved a thorough review to ensure consistency, coherence, data anonymization, and preparation of the dataset for public access. A detailed description of the dataset, titled “nutri_als_dataset.csv,” is available in the public repository at https://doi.org/10.5281/zenodo.12811144.

### 2.4 Data analysis

The data from the educational pathway was analyzed through descriptive statistics, which allowed the team of researchers to fully explore and describe the relevant properties and characteristics of the dataset. The main resources used were measures of absolute and relative frequency; measures of locality, mean, and median; and measures of dispersion or spread, observing the standard deviation (STD).

**Figure 1:**
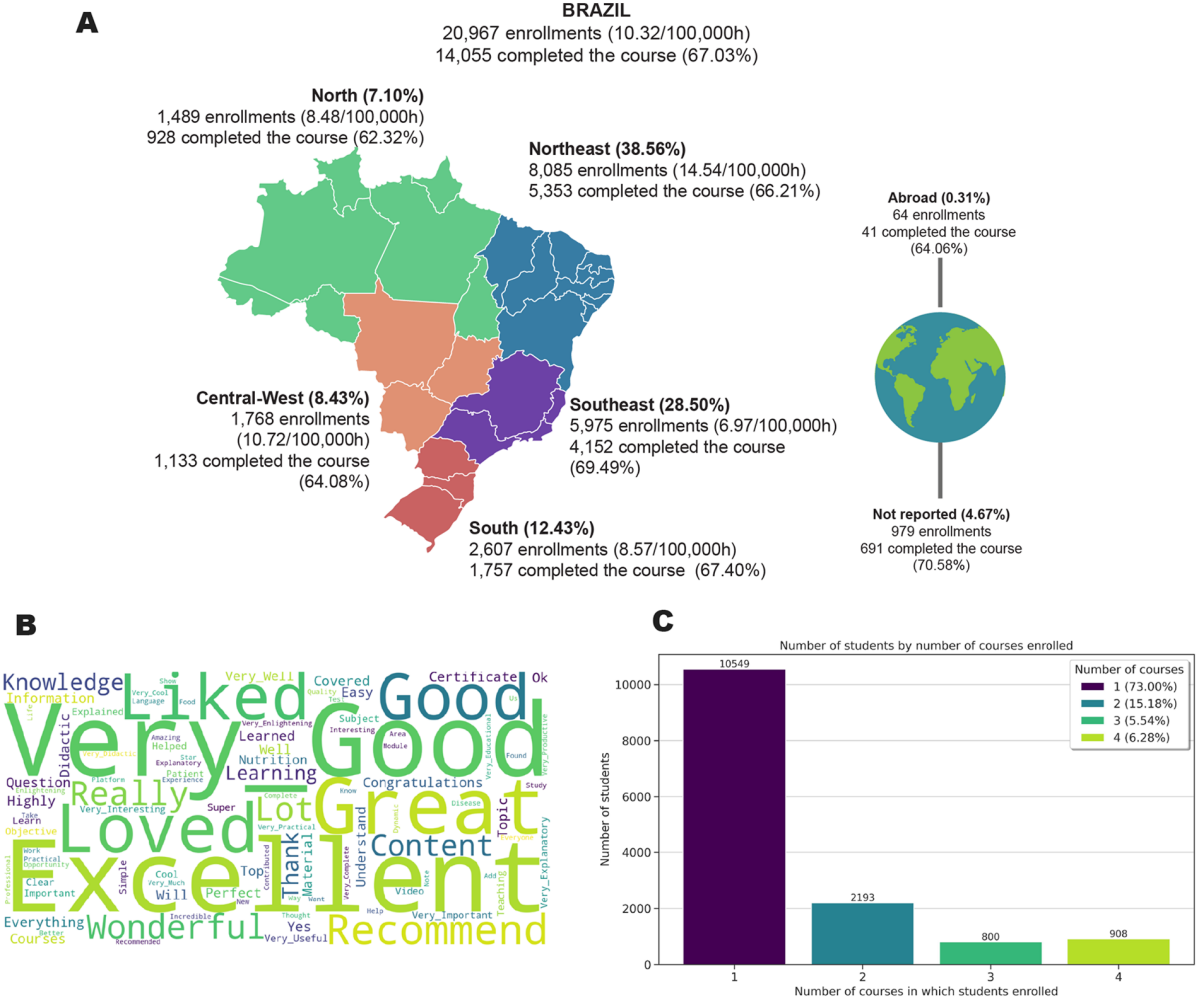
Summary of enrollment and student evaluation data. **(A)** Enrollment distribution across Brazil. **(B)** Word cloud based on students' comments. **(C)** Histogram showing the number of courses enrolled by students.

According to the analysis model proposed by Valentim et al. (32), Equation 1 was necessary to normalize the data related to enrollments and populations in each Brazilian region. Therefore, the variable called “rate” represents the proportion of each analyzed region (normalized values per 100,000 populations). Equation 1 was mainly used to design Figure 1A. The population statistics for Brazil's regions, as well as the country's total population, were retrieved from the IBGE's 2022 demographic census (22). The following notations were defined for the variables in Equation 1:

(1) $$rate\;=\;\left(\frac{x_{target}}{x_{\;pop}}\right)\cdot n_{\;factor}\;\;\;\;\;(1)$$

where,

- rate: variable to store the coefficient for the indicators proportional to each region or to Brazil as whol e
- $x_{target}$: variable to determine the value associated with the indicators related to enrollment numbers
- $x_{\;pop}$: variable to determine the population value for each region
- $n_{\;factor}$: variable to determine the proportionality factor

## 3 Descriptive analysis

**Figure 2:**
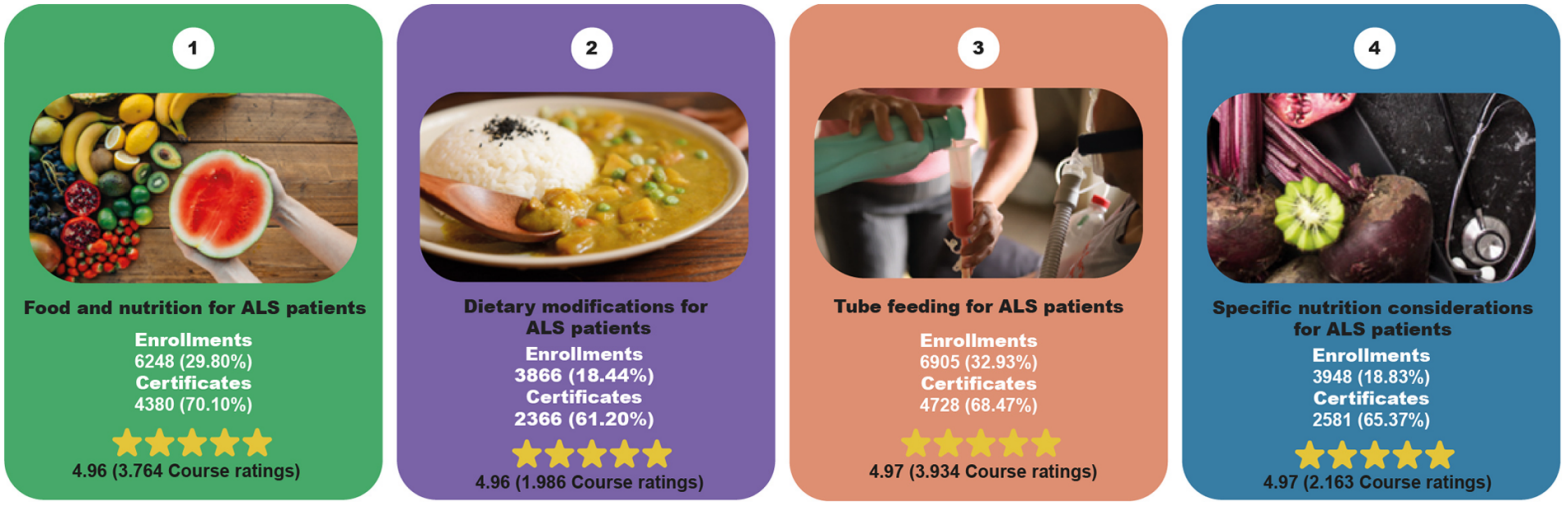
Overview of the educational pathway.

During the study period, the educational pathway on food and nutrition in ALS had a total of 14,450 students, including 3,511 females (24.3%), and 969 males (6.7%). A total of 9,970 students (69%) did not specify their gender. These students made 20,967 enrollments across the four courses offered by the educational pathway. Figure 2 shows the enrollment distribution by course and the number of students who received certificates of completion. To qualify for a certificate, students had to complete 100% of the course activities and achieve a minimum average score of 70%. Overall, 14,055 (97.3%) students were eligible for certificates of completion, averaging approximately 3,514 students per course (median = 3,480, STD = 1,213, quartile 1 at 25th percentile = 2,527, and quartile 2 at 75th percentile = 4,467).

The course entitled “Tube feeding for ALS patients” (https://avasus.ufrn.br/local/avasplugin/cursos/curso.php?id=496), available on AVASUS since July 13, 2021, had the highest number of enrollments among the courses offered in the pathway, with a total of 6,905 enrollments (32.93%). Of these, 4,728 (68.47%) met the criteria to receive a certificate of completion. Additionally, 2,177 (31.53%) students were still in the process of completing the content and activities and were not yet earned the certificate.

The second most popular course in terms of enrollments was “Food and nutrition for ALS patients” (https://avasus.ufrn.br/local/avasplugin/cursos/curso.php?id=492), which has been available since June 1, 2021. This course had 6,248 enrollments (29.80%), and 4,380 students (70.10%) successfully earned a certificate of completion.

Following the analysis of total enrollments, the course “Specific nutrition considerations for ALS patients” (https://avasus.ufrn.br/local/avasplugin/cursos/curso.php?id=497), available since July 13, 2021, had 3,948 enrollments (18.83%). Of these, 2,580 students (65.37%) were eligible to receive a certificate of completion.

Finally, the course “Dietary modifications for ALS patients” (https://avasus.ufrn.br/local/avasplugin/cursos/curso.php?id=493), available since June 1, 2021, received 3,866 (18.44%) enrollments. Of these, 2,366 students (61.20%) were eligible to receive a certificate of completion.

Overall, the technology-mediated self-instructional model used was successful in training the enrolled students, with health professionals representing a subset of the broader participant group. This is evident from the high enrollment numbers and the large number of certificates issued upon completion. On average, 66.28% of enrollments resulted in certificates (median = 66.92%, STD = 3.92%, quartile 1 at 25th percentile = 64.33%, and quartile 2 at 75th percentile = 68.88%).

The enrollments in the educational pathway showed students from all five Brazilian regions as well as from abroad (Figure 1A). Notably, the Northeast Region of Brazil had the highest level of engagement, accounting for 8,085 (38.56%) enrollments. This represents a rate of 14.54 enrollments per 100,000 people. There were also 64 enrollments (0.31%) from international students, with approximately 64% of these students earning a certificate of completion.

At the end of each course, students could rate them using a Likert scale from 1 to 5 stars, with 5 representing the highest level of satisfaction. A total of 11,847 (56.50%) ratings were calculated. The average rating was 4.97 stars (median = 5, STD = 0.27, quartile 1 at 25th percentile = 5, and quartile 2 at 75th percentile = 5). This indicates an excellent level of student satisfaction. The course titled “Tube feeding for ALS patients” received the highest number of evaluations, with 3,934 responses (33.21%). It also achieved an average rating of 4.97 stars (median = 5, STD = 0.25, quartile 1 at 25th percentile = 5, and quartile 2 at 75th percentile = 5).

At the end of each course, students were invited to provide comments. To have a visual representation of the most frequently occurring words and quick feedback from the students, a word cloud analysis was conducted. Out of 20,967 enrollments in the pathway, 4,402 (20.99%) students provided comments about the courses. The word cloud analysis revealed positive feedback from students, indicating favorable evaluation of the courses and a great overall experience (Figure 1B).

The analysis revealed that 908 students (6.28%) completed all four courses offered in the educational pathway, while 10,549 students (73%) enrolled in only one course (Figure 1C). Among the 14,450 students enrolled in the educational pathway, 5,336 (36.93%) were primarily health professionals. Within this group, the following sub-groups were identified: Nursing technicians/aides (1,664–31.18%); Doctors (949–17.78%); Nutritionists (823–15.42%); Nurses (500%–9.37%); and Community Health Workers (475%–8.90%). This distribution is significant, highlighting the importance of multidisciplinary care for ALS patients, as health education is crucial for enhancing care and improving patients’ quality of life.

## Data Availability

The dataset used in this study is available in an online public repository at https://doi.org/10.5281/zenodo.12811144.
